# Production of Carbon Sources Through Anaerobic Fermentation Using the Liquid Phase of Food Waste Three-Phase Separation: Influencing Factors and Microbial Community Structure

**DOI:** 10.3390/bioengineering13010060

**Published:** 2026-01-05

**Authors:** Yangqing Hu, Enwei Lin, Xianming Weng, Fei Wang, Zhenghui Chen, Guojun Lv

**Affiliations:** 1 Fair Friend Institute of Intelligent Manufacturing, Hangzhou Polytechnic University, Hangzhou 310018, China; 2 State Key Laboratory of Clean Energy Utilization, Zhejiang University, Hangzhou 310027, China; 3 Wenzhou Environmental Protection Technology Group Co., Ltd., Wenzhou 325024, China

**Keywords:** resource recycling, microbial treatment, denitrification

## Abstract

The urgent need for effective food waste management, coupled with the scarcity of carbon sources for sewage treatment, highlights the potential of producing carbon sources from food waste as a mutually beneficial solution. This study investigated the production of carbon sources through anaerobic fermentation using the liquid phase of food waste three-phase separation. Compared with previous studies using raw food waste or mixed substrates, the liquid phase derived from three-phase separation is richer in soluble organic matter and has been pre-heated (80 °C), which facilitates subsequent fermentation and offers easier integration into existing food waste treatment plants. A series of lab-scale batch fermentation experiments were carried out at different temperatures, including ambient, mesophilic, and thermophilic conditions, as well as varying initial pH levels (uncontrolled, neutral, and alkaline). The experimental results indicated that optimal production parameters involve a 4-day mesophilic fermentation at 35 °C with an initial alkaline pH, which increased the total VFAs yield by 252.5% to 40.26 g/L and raised the acetic acid fraction to 45.5% of total VFAs. Under these conditions, there was an observed increase in the relative abundance of acidogenic bacteria and a decrease in that of methanogen archaea. Furthermore, the denitrification performance of the produced carbon source was evaluated in short-term tests, and near-complete nitrate removal was achieved within approximately 2 h. These findings suggest the fermented liquid phase of food waste is a promising partial substitute for conventional external carbon sources.

## 1. Introduction

Globally, approximately 1.3 billion tons of food are wasted or lost annually, equivalent to one-third of total human food consumption worldwide [1]. Food waste constitutes about 40% to 60% of municipal solid waste and primarily consists of fats, proteins, and carbohydrates [2]. Inappropriate disposal of food waste releases nutrients such as organic matter, nitrogen, and phosphorus, along with odors, which contribute to secondary pollution [3]. Currently, anaerobic fermentation stands out as one of the principal techniques for recovering energy from food waste. During this process, organic matter is initially converted into intermediates like volatile fatty acids (VFAs), which subsequently leads to biogas generation [4]. In the meantime, a significant challenge in achieving high nitrogen removal efficiency during sewage treatment is attributed to inadequate available carbon sources [5]. Consequently, sewage treatment plants often resort to adding costly external carbon sources such as sodium acetate, methanol, and glucose [6]. Therefore, optimizing anaerobic fermentation processes to enhance VFAs production represents a promising avenue for addressing both energy recovery from food waste and improving sewage treatment efficiency.

Many studies indicate that VFAs carbon source derived from food waste is available for sewage treatment. Zhang et al. [7] generated a carbon source through acidogenic fermentation of food waste, achieving a denitrification rate comparable to that of sodium acetate and exceeding that of methanol. Qi et al. [8] compared the fermentation liquid from food waste with a traditional carbon source, sodium acetate, demonstrating nitrogen removal efficiencies of 87.4% and 95%, respectively. Research on the utilization patterns of VFAs in the denitrification reactions within sewage has revealed a hierarchy in effectiveness—acetic acid > butyric acid > valeric acid > propionic acid [9]. Furthermore, the production and composition of VFAs are closely linked to factors such as temperature, pH, and hydraulic retention time (HRT) [10]. Jiang et al. [11] reported achieving the highest VFAs yields from food waste under mesophilic conditions at a pH of 6. Feng et al. [12] demonstrated that optimal co-fermentation conditions for maximizing VFAs yield from food waste and sludge occurred at pH 8 under ambient temperature. Wu et al. [13] utilized both food waste and sludge for co-fermentation with an optimal HRT of seven days while maintaining an uncontrolled pH range between 5.2 and 6.4; this resulted in hydrolysis and acidification rates reaching 63% and 83.5%, respectively. Cheah et al. [14] reported that compared to the VFAs yield observed at pH 6, the VFAs yield at pH 9 was significantly higher, as was the proportion of acetic acid.

Due to the unique characteristics of food waste in China, specifically its high water and oil content, a food waste treatment plant typically involves sorting, crushing, and subsequently processing the waste through a three-phase separator. This separation yields crude oil, liquid phase, and solid residue. The crude oil is utilized for biodiesel production; the solid residue contains insoluble organic matter and is primarily employed for incineration or composting; while the liquid phase undergoes anaerobic digestion to generate biogas, which further produces electricity to sustain plant operations [15]. Previous research on fermentation substrates for VFAs carbon source production has predominantly focused on food waste or mixtures of food waste with other types of waste (e.g., sludge). In contrast, the liquid phase obtained from three-phase separation is abundant in soluble organic matter, facilitating easier conversion and utilization [16]. Considering factors such as added value, storage capabilities, and transportation logistics, the VFAs carbon source derived from this liquid phase presents a more attractive potential as products of anaerobic fermentation compared to gaseous byproducts [10]. Therefore, provided that normal plant operations are maintained, it is promising to utilize a portion of the liquid phase for VFAs carbon source production.

In this study, the liquid phase of food waste three-phase separation was utilized to produce a VFAs carbon source through anaerobic fermentation. The influencing factors, including temperature, pH, and HRT, were thoroughly researched. Additionally, shifts in microbial community structure were examined to identify the predominant bacterial species present during liquid phase fermentation under various conditions and to analyze the correlations between microbial communities and VFAs production. Furthermore, the denitrification performance of the produced VFAs carbon source was evaluated. Compared with previous research that primarily used raw food waste or co-substrates such as sludge [7,10,13], this study focuses specifically on the liquid phase from three-phase separation—a byproduct readily available in full-scale treatment plants. This approach not only leverages the pre-heated, soluble-rich characteristics of the liquid phase for more efficient VFAs conversion, but also presents practical advantages in terms of direct integration into existing treatment lines, reduced handling requirements, and enhanced storage and transport potential.

## 2. Materials and Methods

### 2.1. Materials and Reagents Used

The substrate used in this study was sourced from a food waste treatment plant located in Wenzhou, China. The operational procedure of the plant includes sorting, crushing, three-phase separation (at 80 °C), anaerobic digestion, biogas purification, and power generation. Samples were collected from the liquid phase of the three-phase separation process. The inoculum used for fermentation consisted of municipal sludge obtained from a sewage treatment plant in Shanghai, China. The appearance of samples is demonstrated in Supplementary Material Figure S1. Glucose, peptone, yeas, L-cysteine, K_2_HPO_4_, KH_2_PO_4_, MgSO_4_, FeSO_4_·7H_2_O, NiCl_2_·6H_2_O, CuSO_4_·5H_2_O, CoCl_2_·5H_2_O, MnSO_4_·6H_2_O, and ZnCl_2_ were used as nutrient solutions for cultivating inoculum [17].

### 2.2. Anaerobic Fermentation Experiments

The experiments were conducted using batch fermentation devices. A series of 500 mL sealed bottles were placed in a constant-temperature water bath. Compared to a low substrate-to-inoculum ratio, a high substrate-to-inoculum ratio leads to elevation of the VFAs concentration over the anaerobic digestion period [18]. Also, referring to the research of other scholars [19], the substrate and inoculum were introduced into the reactor at a ratio of 3:1 in this study. To ensure a consistent temperature was maintained throughout the fermentation process, the devices were placed in a constant-temperature water bath. The influence of temperature was investigated by maintaining the reactor at ambient temperature (approximately 25 °C), mesophilic temperature (35 °C), and thermophilic temperature (55 °C). The effect of pH was examined by setting the initial pH to neutral (7.0), alkaline (9.0), and uncontrolled conditions. To enhance the reproducibility of the experimental results, three reactors were established under each condition, with average values recorded for analysis. The experiments spanned a duration of 10 days until VFAs reached stability, during which samples were extracted daily for further tests.

### 2.3. Denitrification Experiments

To evaluate the denitrification performance of the produced carbon source, sewage and denitrification sludge from a sewage treatment plant were introduced into a series of reactors equipped with continuous agitators [8], as Figure S2 shows. The characteristics of sludge during denitrification are listed in Table S1. Effluent samples were collected every 30 min for the analysis of inorganic nitrogen ($NO_{3}^{-}-N$ and $NO_{2}^{-}-N$).

### 2.4. Physicochemical Analysis

TS and VS were measured following standard methods [20]. The pH was determined using a pH meter (Mettler FE28, Zurich, Switzerland). Inorganic nitrogen levels were assessed with an ultraviolet spectrophotometer (Shimadzu UV2600, Kyoto, Japan). A gas chromatograph (Shimadzu GC 2014 C, Kyoto, Japan) equipped with an AT-FFAP column (30 m in length, 0.32 mm internal diameter, and 0.5 μm film thickness) and a flame ionization detector (FID) was employed to quantify the concentrations of various VFAs. Nitrogen served as the carrier gas at a flow rate of 50 mL/min. The injector and detector temperatures were maintained at 200 °C and 230 °C, respectively. The oven temperature program commenced at 110 °C for two minutes before increasing to 190 °C at a rate of 10 °C per minute, followed by an additional two-minute holding period. A sample injection volume of 1.0 µL was utilized [21].

### 2.5. Microbial Community Analysis

Total microbial genomic DNA was extracted from samples using the E.Z.N.A.^®^ soil DNA Kit (Omega Bio-tek, Norcross, GA, USA) according to the manufacturer’s instructions. The quality and concentration of DNA were determined by 1.0% agarose gel electrophoresis and a NanoDrop2000 spectrophotometer (Thermo Scientific, Waltham, MA, USA) and kept at −80 °C prior to further use. For bacterial polymerase chain reaction (PCR) amplification, the primers 338F (ACTCCTACGGGAGGCAGCAG) and 806R (GGACTACHVGGGTWTCTAAT) were used, whereas for the archaeal sequence library, the primers 344F (ACGGGGYGCAGCAGGCGCGA) and 915R (GTGCTCCCCCGCCAATTCCT) were used. Purified amplicons were pooled in equimolar amounts and paired-end sequenced on an Illumina Nextseq2000 platform (Illumina, San Diego, CA, USA) according to the standard protocols.

## 3. Results and Discussion

### 3.1. Substrate and Inoculum Properties

The substrate samples were collected from the liquid phase of the three-phase separation process. The total solids (TS) and volatile solids (VS) content of the substrate were measured at 6.8% and 6.3%, respectively. Additionally, the pH of the substrate was determined to be 4.34. During the high-temperature three-phase separation process, macromolecular organic substances were converted into micromolecular organic compounds, such as VFAs. The total concentration of VFAs in the substrate was found to be 11.42 g/L, with acetic acid accounting for 5.46 g/L, propionic acid for 1.71 g/L, and butyric acid for 4.15 g/L—together constituting the majority. The inoculum was sample-collected from municipal sludge of a sewage treatment plant. The TS and VS, content and pH level of this inoculum were recorded at 3.96%, 3.67%, and 5.86, respectively.

### 3.2. Effect of Temperature on Carbon Source Production

#### 3.2.1. VFAs’ Production and Composition Under Different Temperatures

**Figure 1:**
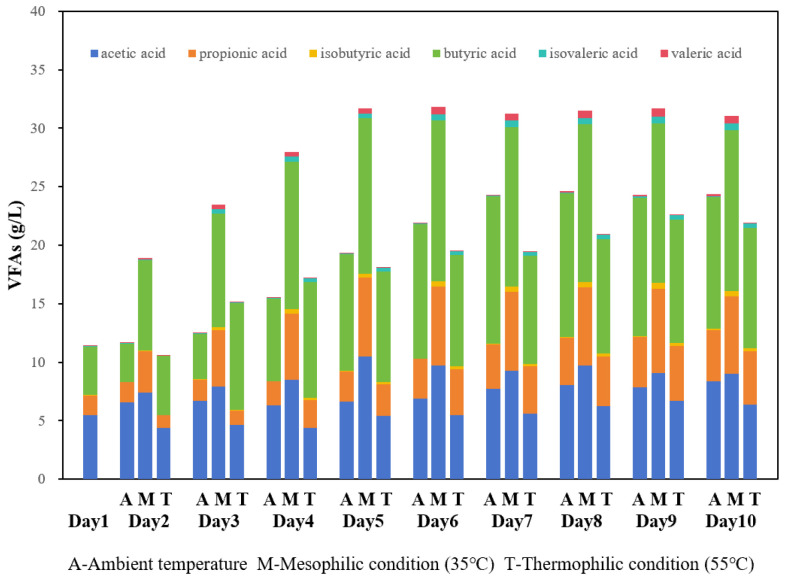
VFAs concentration under different temperature conditions: ambient, mesophilic (35 °C), and thermophilic (55 °C).

Temperature is a critical factor influencing anaerobic fermentation. The optimal temperature range for anaerobic bacteria primarily lies within the mesophilic (35 °C) and thermophilic (55 °C) categories. To investigate the impact of temperature, three sets of experiments were conducted at different temperatures: ambient, mesophilic, and thermophilic. The results indicated that mesophilic conditions were optimal, yielding a stable total VFA concentration of 31.72 g/L after 5 days (Figure 1). This represented a 177.8% increase from the initial concentration. Both ambient and thermophilic fermentation also increased VFA levels, but the effects were less pronounced than those at 35 °C. Ambient temperature is generally unsuitable for the activity of anaerobic microorganisms, which negatively impacts VFAs production. In contrast, higher temperatures during thermophilic anaerobic fermentation can effectively enhance the hydrolysis stage, thereby increasing yields of fermentation products [22]. However, the substrate used in this study had already been subjected to heating at 80 °C during the three-phase separation process, thus limiting the additional benefits from high-temperature hydrolysis. Furthermore, acidogenic bacteria tend to favor mesophilic environments [23], which explains why VFAs yields at thermophilic temperatures are suboptimal.

**Figure 2:**
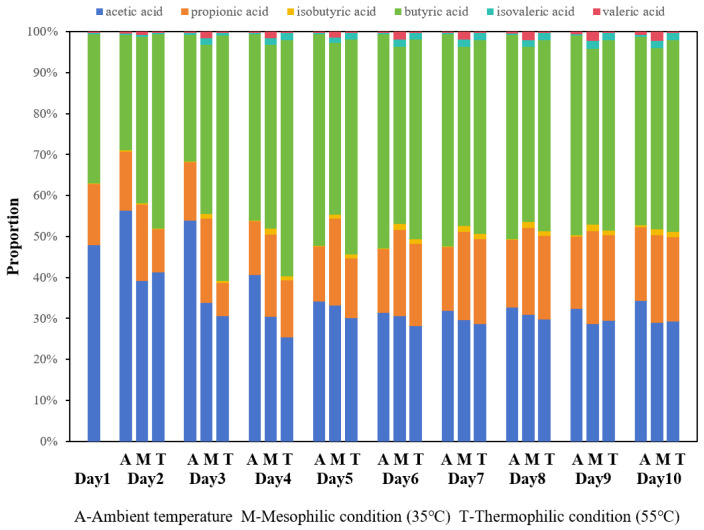
Distribution of VFAs of fermentation under different temperature conditions: ambient, mesophilic (35 °C), and thermophilic (55 °C).

The distribution of individual VFAs also varied with temperature. After stabilization (day 5), butyric acid was the most abundant, followed by acetic and propionic acid across all temperatures (Figure 2). Under mesophilic conditions, the proportion of acetic acid was higher than under thermophilic conditions, though still lower than that of butyric acid. Notably, during mesophilic fermentation, the acetic acid proportion decreased while that of butyric acid increased gradually. This observation suggested that produced butyric acid was not rapidly converted into acetic acid during the acetogenesis stage or that some portion of acetic acid underwent further conversion into methane during methanogenesis, leading to a reduction in the proportion of acetic acid.

#### 3.2.2. Microbial Community Structure Under Different Temperatures

**Figure 3:**
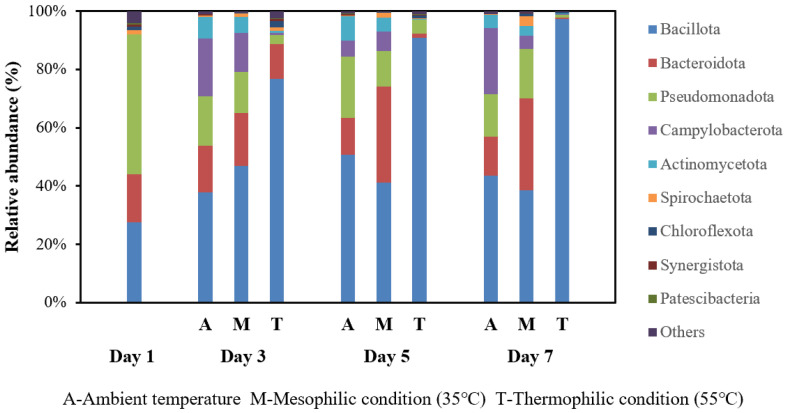
Temporal evolution of bacterial community composition at the phylum level during fermentation under different temperature conditions: ambient, mesophilic (35 °C), and thermophilic (55 °C).

Microbial community analysis provided insights into the varying VFA yields. At the phylum level, *Bacillota*, *Bacteroidota*, *Pseudomonadota*, and *Campylobacterota* were dominant (Figure 3). *Bacillota*, also known as *Firmacutes* [24], is considered to be a fermenter and syntrophic bacteria [25], and is capable of producing extracellular enzymes that play a crucial role in the biodegradation of macromolecular organic matters [26]. *Bacteroidota*, a correction of the effectively published synonym *Bacteroidetes* [24], includes many functional bacteria, such as hydrolytic and acidogenic bacteria [27]. The most significant contribution of *Bacteroidota* to waste processing is the hydrolysis of carbohydrates into VFAs [28]. *Pseudomonadota*, a correction of the effectively published synonym *Proteobacteria* [24], has been regarded as a functional phylum in the anaerobic fermentation of the organics [29]. *Campylobacterota*, also known as *Epsilonbacteraeota* [30], is an important chemolithotrophic primary producer and performs sulfur oxidation coupled with N-oxide reduction while fixing carbon [30]. At mesophilic temperatures, the relative abundance of *Bacteroidota* and *Bacillota* increased rapidly in the initial days of fermentation, which coincided temporally with the observed higher VFAs production. The greater community diversity may have collectively contributed to better VFAs production. However, this association requires deeper functional analysis to confirm the specific roles of individual populations. In contrast, the thermophilic bacterial community was less diverse, dominated by *Bacillota* with *Bacteroidota* being almost absent, which may explain the suboptimal VFAs yield. At ambient temperature, lower VFAs production could hypothetically be due to the weaker activity of functional bacteria, potentially leading to a decrease in VFAs generation efficiency and a longer stabilization time (7 days vs. 5 days).

**Figure 4:**
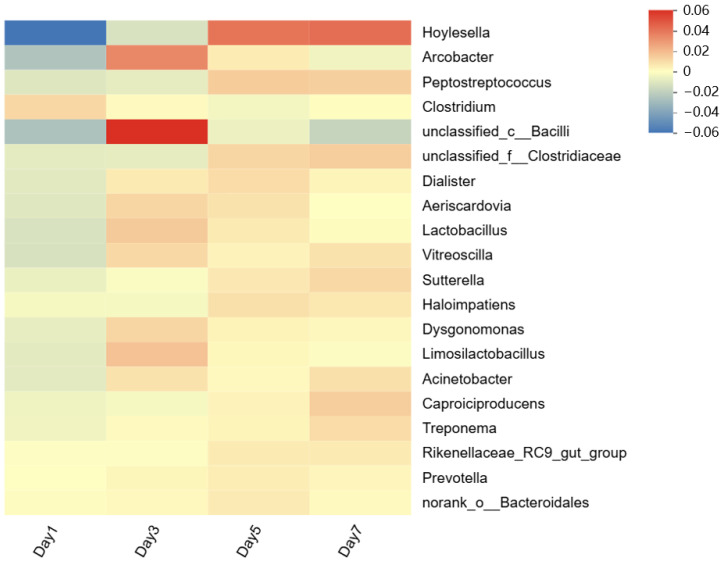
Heatmap showing the evolution of the relative abundance of the top 20 bacterial genera during mesophilic fermentation (35 °C).

It is important to note that a bacterial phylum often consists of multiple members at lower levels, each of which may display distinct phenotypes and functions [31]. To further investigate the functional bacteria at mesophilic temperature, 20 main genera were selected to draw a heatmap, as shown in Figure 4. The heatmap revealed that the relative abundance of *Hoylesella* and *Peptostreptococcus* increased during the fermentation process. Various species within the genus *Hoylesella*, which belongs to the phylum *Bacteroidota*, are able to ferment organic matter (e.g., glucose, lactose and maltose) [32]. Similarly, the genus *Peptostreptococcus*, which belongs to the phylum *Bacillota*, produces acetic, butyric, propionic, isobutyric, and isovaleric acids as its main metabolic products [33]. The results showed that bacteria involved in the decomposition of macromolecular organics and the production of acid are crucial for the generation of VFAs carbon sources.

### 3.3. Effect of Initial pH in Carbon Source Production

#### 3.3.1. VFAs’ Production and Composition Under Different Initial pH Conditions

**Figure 5:**
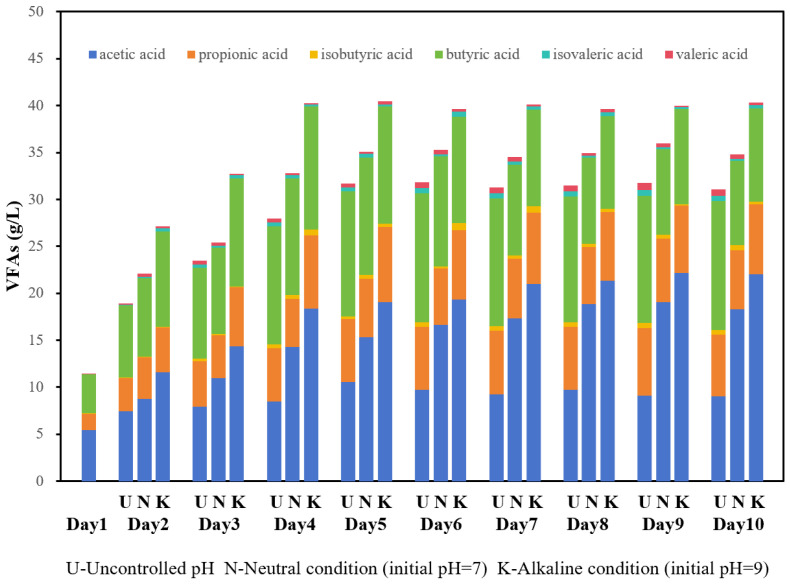
VFAs concentration during mesophilic fermentation (35 °C) under different initial pH conditions: uncontrolled, neutral (pH 7.0), and alkaline (pH 9.0).

pH was another critical factor. Under mesophilic temperature (35 °C), the alkaline group (with an initial pH of 9) achieved a stable VFAs concentration one day earlier (day 4) than the neutral (pH 7) and uncontrolled pH groups (Figure 5). When the VFAs concentrations across all groups reached a near-stable state, on the fourth day for the initial alkaline group and on the fifth day for both neutral and uncontrolled groups, the total concentrations of VFAs in both alkaline and neutral groups increased by 26.9% and 10.6%, respectively, compared to those in the uncontrolled group. The VFAs concentration of the alkaline group reached a total amount of 40.26 g/L, reflecting an increase of 252.5% compared to the initial concentration. These variations were associated with differing bacterial community adaptations to pH levels, which corresponded to notable differences in VFAs concentrations. Recent research indicated that a lower pH obviously inhabits the production of VFAs [34,35]. In contrast, alkaline conditions are generally considered to enhanced acidogenic bacterial activity while inhibiting methanogen activity [36], which may be one mechanism underlying the enhanced VFA production observed under alkaline conditions in this study.

**Figure 6:**
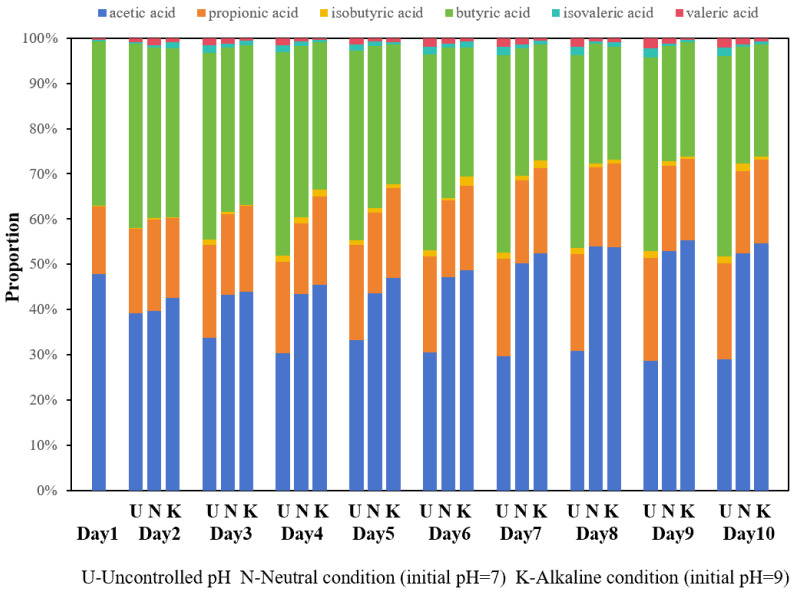
Distribution of VFAs of mesophilic fermentation (35 °C) under different initial pH conditions: uncontrolled, neutral (pH 7.0), and alkaline (pH 9.0).

The VFAs distribution was also affected (Figure 6). The results demonstrated that the proportion of acetic acid in the initial alkaline group gradually increased, while the proportion of butyric acid decreased. This phenomenon may be attributed to the inhibition of methanogen activity, which hindered further conversion of acetic acid into methane, resulting in an accumulation of acetic acid. Furthermore, the proportion of acetic acid in the initial alkaline group was found to be higher than that in other groups. According to the utilization hierarchy of VFAs (acetic acid > butyric acid > valeric acid > propionic acid) relevant to sewage denitrification performance [9], both the total quantity and quality of VFAs carbon sources produced through anaerobic fermentation in the initial alkaline group were superior compared to those from the other two groups. Therefore, it is recommended that a 4-day mesophilic fermentation with an initial alkaline pH serves as an optimal parameter for VFAs carbon source production.

**Figure 7:**
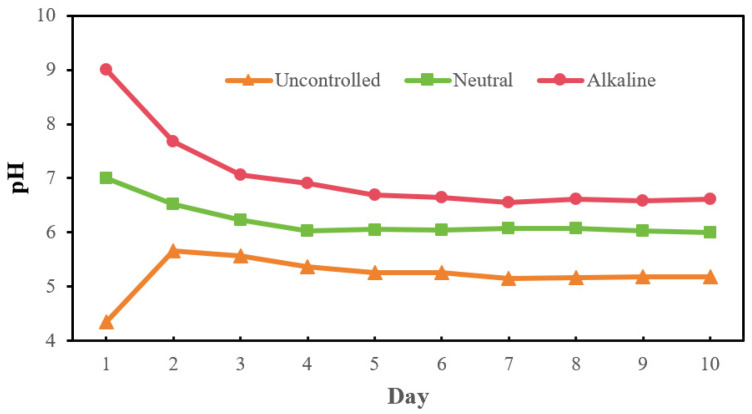
Profile of pH change over time during mesophilic fermentation (35 °C) initiated at different pH levels: uncontrolled, neutral (pH 7.0), and alkaline (pH 9.0).

The pH dynamics varied by group (Figure 7). In the initial alkaline group, pH declined due to rapid production of VFAs during the first few days and subsequently stabilized at around 7 during the following days. Similarly, in the initial neutral group, the pH also exhibited an initial decline before stabilizing at approximately 6. Conversely, in the uncontrolled group, the pH initially increased. This phenomenon can be attributed to a potential scenario in which the optimal pH for lactic acid production is around 4.5 [37], resulting in a rapid accumulation of lactic acid at first. Furthermore, lactic acid served as a suitable electron donor for chain elongation processes that produce medium-chain fatty acids; this consumption of H^+^ during these reactions could lead to an increase in pH [38]. Eventually, pH tended toward stabilization.

#### 3.3.2. Microbial Community Structure Under Different Initial pH Conditions

**Figure 8:**
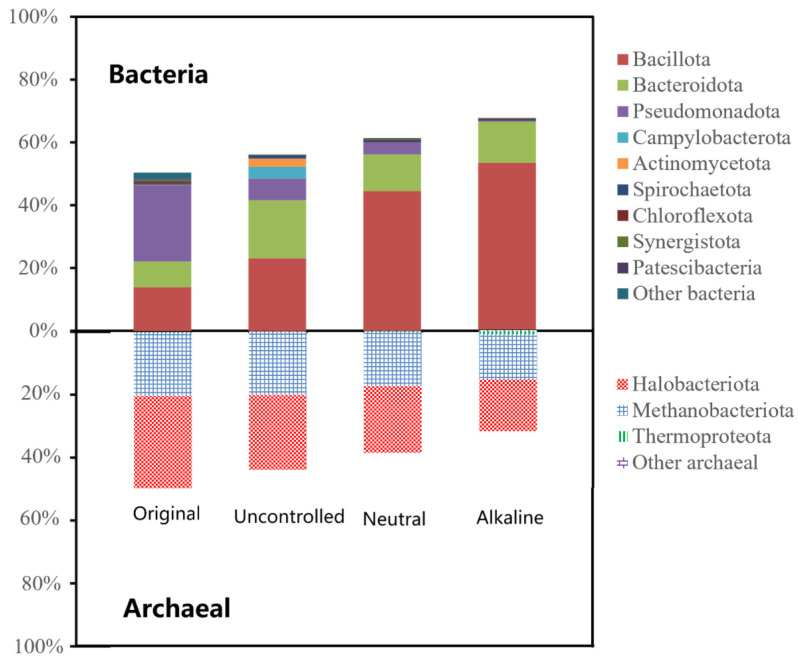
Relative abundance of dominant bacterial and archaeal phyla after 5 days of mesophilic fermentation (35 °C) under different initial pH conditions: uncontrolled, neutral (pH 7.0), and alkaline (pH 9.0).

The microbial community structure after 5 days of fermentation clearly differed with the initial pH (Figure 8). As the initial pH increased, the proportion of bacteria rose while that of archaea declined. The functions of the main bacteria are introduced above. The dominant archaeal phyla were *Halobacteriota* and *Methanobacteriota*. *Halobacteriota*, a new higher taxa based on the nomenclature types of the valid genus *Halobacterium* [39], grows anaerobically by fermentation of arginine [40]. *Methanobacteriota* is located at the end of the anaerobic digestion food chain and is strictly made up of anaerobic microorganisms [41]. *Methanobacteriota* is able to produce methane by utilizing simple compounds such as acetic acid, hydrogen/carbon dioxide, and methyl compounds [42]. It is commonly hypothesized that a high proportion of methanogenic archaea can rapidly consume acetic acid, potentially leading to a decrease in VFAs yield. In this study, the lower acetic acid proportion observed under non-alkaline conditions is consistent with this view. Under alkaline conditions, the observed high relative abundance of acidogenic bacteria and low relative abundance of methanogenic archaea were associated with the optimal VFAs production. This community profile likely collectively supported the optimal production of VFAs.

### 3.4. Denitrification Performance

**Figure 9:**
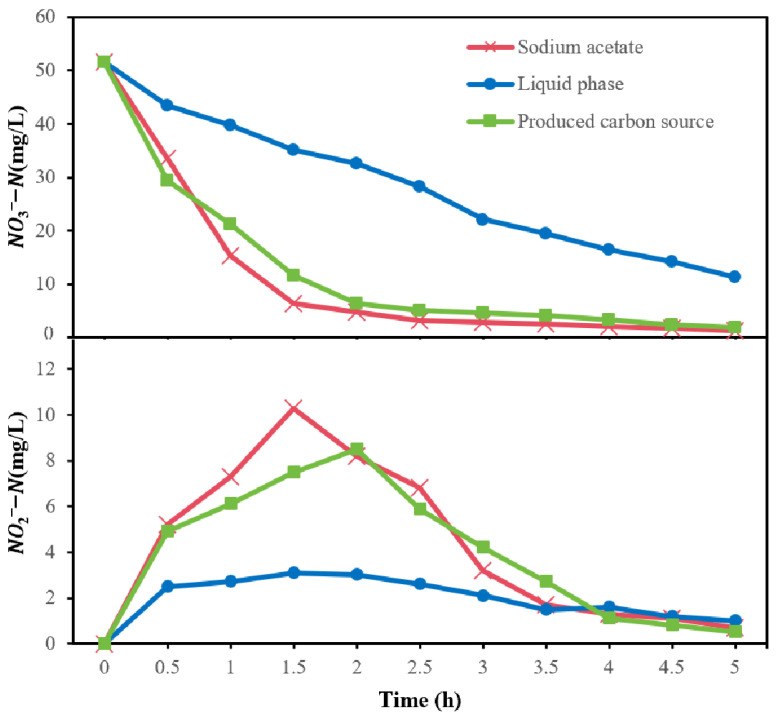
Denitrification performance of different carbon sources: sodium acetate (traditional carbon source), raw three-phase separation liquid phase, and the produced VFA carbon source (from 4-day mesophilic fermentation at initial pH 9).

The denitrification performance of the produced carbon source, derived from a 4-day mesophilic fermentation with an initial alkaline pH, was compared to that of traditional carbon sources (sodium acetate) and untreated fermentation substrates (the liquid phase of three-phase separation), as illustrated in Figure 9. The concentration of $NO_{3}^{-}-N$ exhibited a gradual decrease over time. Notably, the decline rate observed in the liquid phase group was lower than that in the other groups. This can be attributed to the limited percentage of micromolecular organic matter present in the liquid phase, resulting in a relatively low utilization rate for denitrification [43]. In contrast, sodium acetate is recognized as an ideal biodegradable micromolecular organic compound for denitrification purposes; consequently, the $NO_{3}^{-}-N$ concentration within this group decreased rapidly and remained below 5 mg/L after 1.5 h. The produced carbon source contained a high concentration of VFAs, which can be readily utilized by denitrifying bacterial communities. The decline curve for $NO_{3}^{-}-N$ associated with the produced carbon source closely mirrored that of sodium acetate; however, it took approximately 2 h to achieve similarly low concentrations of $NO_{3}^{-}-N$.

Initially, there was an increase in $NO_{2}^{-}-N$ concentration due to the conversion from $NO_{3}^{-}-N$ to $NO_{2}^{-}-N$. Subsequently, this concentration decreased as a result of further conversion from $NO_{2}^{-}-N$ to N_2_ [44]. When compared with sodium acetate, the peak cumulative concentration of $NO_{2}^{-}-N$ within the produced carbon source group was lower. Additionally, because reaction rates were slower within the liquid phase group, fluctuations in $NO_{2}^{-}-N$ concentrations were less pronounced than those observed in other groups.

To further assess the engineering relevance of the produced carbon source, a basic quantitative comparison was conducted. Assuming a typical municipal wastewater treatment plant with a flow of 100,000 m^3^/d and a need to remove 30 mg/L of nitrate nitrogen, the daily external carbon demand (as COD) would be roughly 18,000 kg COD/d (using a C/N ratio of 6). The fermentation process described in this study, when applied to the liquid phase from a food waste treatment plant with a capacity of 200 tons/d (assuming ~80% liquid yield and VFAs-COD concentration of 57.2 g equivalent-COD/L), could theoretically yield approximately 9000 kg VFA-COD per day. This suggests that the fermented liquid from such a source could potentially supply around 50% of the external carbon demand for the wastewater treatment plant, demonstrating its significant potential as a supplemental carbon source. It is important to note that this is a preliminary estimation; a detailed techno-economic analysis, considering factors such as transportation, stability, and full-scale process integration, alongside verification in pilot-scale or full-scale systems, constitutes essential future work.

In summary, while demonstrating effective denitrification performance overall, it is evident that produced carbon sources can serve as viable external alternatives to partially replace traditional carbon sources. Recent studies have also shown that VFAs are technically feasible, economically viable, and have considerable prospects [45,46].

## 4. Conclusions

This study investigated the production of carbon sources through anaerobic fermentation using the liquid phase of food waste three-phase separation. Initially, the effect of temperature on VFAs yield and composition was examined. The results indicated that VFAs production under mesophilic conditions outperformed that observed at ambient and thermophilic temperatures. Furthermore, an analysis of the bacterial community structure revealed that the bacteria diversity was richer and the relative abundance of acidogenic functional bacteria was higher at mesophilic temperatures. Subsequently, a comparison of initial pH levels demonstrated that alkaline conditions were advantageous for VFAs production, achieving a 252.5% increase in total VFAs concentration to 40.26 g/L and elevating the acetic acid proportion to 45.5%. The examination of the microbial community structure further illustrated that a high relative abundance of acidogenic bacteria and a low relative abundance of methanogenic archaea led to the optimal production of VFAs under alkaline conditions. In conclusion, we recommend a 4-day mesophilic fermentation process with an initial alkaline pH as optimal parameters for maximizing VFA carbon source production from the liquid phase of food waste three-phase separation. Notably, in short-term denitrification tests, the produced carbon source achieved near-complete nitrate removal within 2 h, demonstrating a performance comparable to sodium acetate. This positions the fermented liquid phase of food waste as a promising partial substitute for conventional carbon sources. It is important to note that this research was conducted under controlled lab-scale batch conditions. Future work should address long-term operational stability, detailed techno-economic assessment, and verification at pilot or full scale.

## Data Availability

The data that support the findings of this study are available on request from the corresponding author.

## Supplementary Materials

The following supporting information can be downloaded at https://www.mdpi.com/article/10.3390/bioengineering13010060/s1, Figure S1: the appearance of samples; Figure S2: Reactor configuration of the denitrification tests; Table S1. The characteristics of sludge in denitrification; Table S2. VFAs concentrations at varying temperatures; Table S3. VFAs concentrations at varying initial pH levels.

## Abbreviations

The following abbreviations are used in this manuscript:

VFAs	Volatile Fatty Acids.
