# Supplementary material for: Production of Carbon Sources Through Anaerobic Fermentation Using the Liquid Phase of Food Waste Three-Phase Separation: Influencing Factors and Microbial Community Structure
Source: Bioengineering (Basel). 2026 Jan 5;13(1):60. doi: 10.3390/bioengineering13010060 (PMC12837757; doi:10.3390/bioengineering13010060)
Supplement: Supplementary file 1 [file bioengineering-13-00060-s001.zip › bioengineering-4060991-supplementary.pdf]

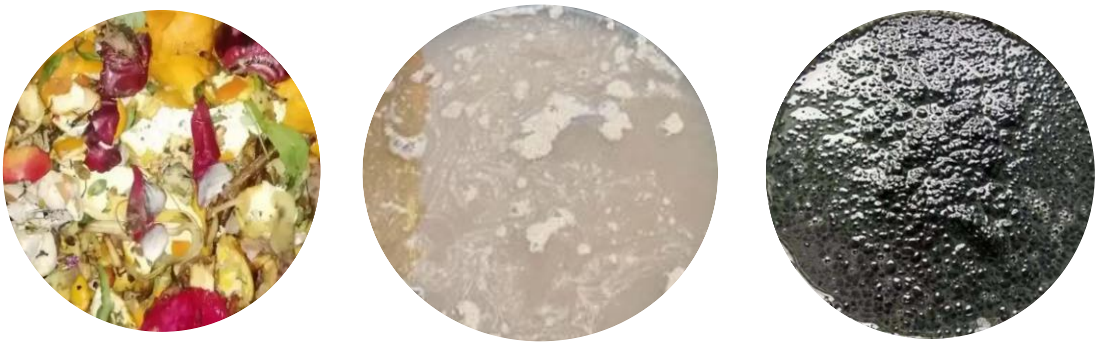

(a) (b) (c)

Figure S1. the appearance of samples (a) food waste (b) liquid phase (c) inoculum sludge

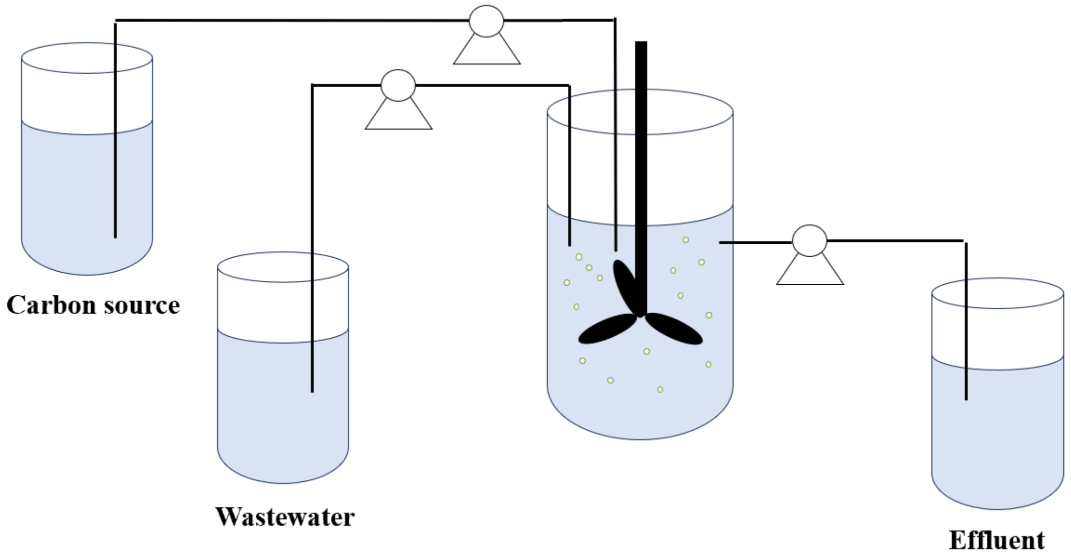

Figure S2. Reactor configuration of the denitrification tests

Table S1. The characteristics of sludge in denitrification

characteristics	sludge
TS (%)	16.2
VS (%)	7.3
pH	5.23

Table S2. VFAs concentrations at varying temperatures

Concentration (g/L)	Day 1	Day 2			Day 3			Day 4			Day 5			A- Ambient temperature
		A	M	T	A	M	T	A	M	T	A	M	T	
acetic acid	5.46	6.59	7.42	4.37	6.73	7.92	4.64	6.32	8.50	4.36	6.61	10.51	5.44	
propionic acid	1.71	1.70	3.52	1.11	1.79	4.82	1.23	2.03	5.64	2.40	2.61	6.72	2.64	M- Mesophilic condition (35℃)
isobutyric acid	0.02	0.02	0.06	0.02	0.02	0.26	0.06	0.02	0.39	0.17	0.03	0.30	0.20	
butyric acid	4.15	3.30	7.69	5.03	3.87	9.69	9.09	7.08	12.56	9.96	10.00	13.31	9.49	
isovaleric acid	0.03	0.05	0.09	0.04	0.04	0.38	0.10	0.04	0.45	0.29	0.06	0.43	0.29	T- Thermophilic condition (55℃)
valeric acid	0.04	0.05	0.15	0.03	0.04	0.38	0.05	0.05	0.43	0.06	0.05	0.44	0.06	

Concentration (g/L)	Day 6			Day 7			Day 8			Day 9			Day 10		
	A	M	T	A	M	T	A	M	T	A	M	T	A	M	T
acetic acid	6.89	9.73	5.50	7.75	9.25	5.58	8.04	9.70	6.24	7.86	9.08	6.68	8.36	9.01	6.40
propionic acid	3.3	6.71	3.91	3.77	6.75	4.04	4.04	6.73	4.26	4.29	7.22	4.71	4.37	6.60	4.52
isobutyric acid	0.03	0.45	0.23	0.04	0.46	0.24	0.05	0.45	0.25	0.08	0.52	0.28	0.11	0.47	0.28
butyric acid	11.48	13.77	9.50	12.61	13.66	9.23	12.28	13.44	9.78	11.84	13.57	10.53	11.23	13.75	10.28
isovaleric acid	0.07	0.55	0.32	0.0	0.54	0.34	0.08	0.55	0.36	0.10	0.62	0.39	0.11	0.57	0.38
valeric acid	0.07	0.61	0.06	0.07	0.61	0.07	0.09	0.62	0.07	0.12	0.72	0.07	0.17	0.66	0.07

Table S3. VFAs concentrations at varying initial pH levels

Concentration (g/L)	Day 1	Day 2			Day 3			Day 4			Day 5			U- Uncontrolled pH N- Neutral condition (initial pH=7) K- Alkaline condition (initial pH=9)
		U	N	K	U	N	K	U	N	K	U	N	K	
acetic acid	5.46	7.42	8.74	11.58	7.92	10.96	14.37	8.50	14.24	18.33	10.51	15.32	19.03	
propionic acid	1.71	3.52	4.45	4.76	4.82	4.55	6.23	5.64	5.13	7.83	6.72	6.22	8.02	
isobutyric acid	0.02	0.06	0.08	0.07	0.26	0.13	0.09	0.39	0.44	0.62	0.30	0.37	0.34	
butyric acid	4.15	7.69	8.33	10.13	9.69	9.21	11.57	12.56	12.41	13.12	13.31	12.57	12.55	
isovaleric acid	0.03	0.09	0.12	0.35	0.38	0.22	0.31	0.45	0.34	0.23	0.43	0.36	0.21	
valeric acid	0.04	0.15	0.33	0.26	0.38	0.31	0.18	0.43	0.24	0.13	0.44	0.25	0.32	

Concentration (g/L)	Day 6			Day 7			Day 8			Day 9			Day 10		
	U	N	K	U	N	K	U	N	K	U	N	K	U	N	K
acetic acid	9.73	16.62	19.33	9.25	17.34	21.01	9.70	18.81	21.33	9.08	19.03	22.12	9.01	18.25	22.03
propionic acid	6.71	5.98	7.37	6.75	6.33	7.58	6.73	6.13	7.35	7.22	6.79	7.19	6.60	6.32	7.47
isobutyric acid	0.45	0.21	0.78	0.46	0.32	0.69	0.45	0.31	0.29	0.52	0.41	0.15	0.47	0.56	0.24
butyric acid	13.77	11.76	11.34	13.66	9.73	10.28	13.44	9.23	9.89	13.57	9.11	10.14	13.75	8.96	9.98
isovaleric acid	0.55	0.25	0.56	0.54	0.31	0.34	0.55	0.16	0.45	0.62	0.23	0.22	0.57	0.19	0.31
valeric acid	0.61	0.45	0.26	0.61	0.46	0.21	0.62	0.26	0.32	0.72	0.42	0.13	0.66	0.49	0.26
